# Development of a low-cost electrospinning system with a bidirectional collector for uniform nanofibrous membranes

**DOI:** 10.1016/j.ohx.2025.e00704

**Published:** 2025-09-17

**Authors:** Jhonatan A. Gutierrez-Rivera, Andres F. Roca-Arroyo, David A. Castilla-Casadiego, Alberto Albis

**Affiliations:** a Department of Biomedical Engineering, University of Miami, Coral Gables, FL 33146, USA; b Chemical Engineering Program, Faculty of Engineering, Universidad del Atlántico, Carrera 30 8-49, Puerto Colombia, Atlántico, Colombia

**Keywords:** Electrospinning, Nanomembranes, Flat plate, Polymeric materials, Polystyrene

## Abstract

The electrospinning process is a widely used technique for the fabrication of membranes with nanometric fibers, employing polymeric materials such as polyvinylidene fluoride and polycaprolactone. The shape of the fiber collector, whether static or rotating, significantly impacts membrane uniformity. Although rotating drum collectors are the most used, they exhibit drawbacks such as uneven fiber accumulation. Current solutions, which favor rotating over static collectors, tend to be more expensive and complex. This article presents an electrospinning setup that utilizes a flat acrylic plate with bidirectional movement along the X and Y axes, enhancing fiber collection and membrane uniformity. This design improves process efficiency, fiber reproducibility, and system scalability. Polystyrene electrospun nanofibrous membranes were fabricated, and their average fiber diameter and pore size were analyzed, demonstrating the system’s capability to produce micro- and nanometric fibers.

## Graphical abstract

**Figure:**
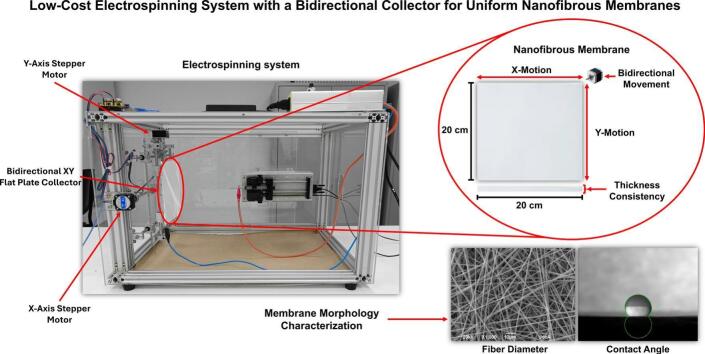

**Specifications table:** 

Hardware name	Electrospinning setup with a flat plate collector with X- and Y-axis motion
Subject area	Engineering and materials science, biomedical materials
Hardware type	Manufacturing
Closest commercial analog	Spinbox (Fluidnatek)
Open-source license	Creative Commons Attribution (CC BY) 4.0 license
Cost of hardware	$ 1546.97
Source file repository	https://doi.org/10.17632/44t3m6yzt9.2

## 1 Hardware in context

The electrospinning process is a widely used technique for manufacturing non-woven membranes with fiber diameters ranging from tens of nanometers to a few micrometers [1]. These membranes can be made from various polymeric materials, including polyvinylidene fluoride (PVDF), which is extensively employed in water treatment processes [2], and polycaprolactone (PCL), commonly used in tissue engineering [3], among others. Electrospinning is performed by applying a potential difference across an electrostatic field established between a syringe containing a polymer solution, which is dispensed through a needle, and a collector made of or coated with electrically conductive material [4,5]. This high voltage generates a fine polymer jet that is stretched, solidified, and deposited onto the collector, forming ultrathin fibers with nanometric thickness [6,7].

The electrospinning technique has gained significant attention in the scientific community due to its versatility, ease of use, and the wide range of applications for the membranes produced [8]. The applications of fibers obtained via electrospinning span diverse fields. These include water treatment and environmental care, through processes such as salt and contaminant separation, membrane distillation, photocatalysis, and chemical and gas detection; energy-related uses include batteries, fuel cells, supercapacitors, and solar cells [[9], [10], [11], [12]]. Additionally, novel applications in human health, such as tissue engineering, wound dressings, drug delivery, therapeutic processes, and biological sensors, have emerged [[13], [14], [15]]. This demonstrates the versatility and simplicity of the electrospinning technique in adapting to various needs [8]. Although the most common method of electrospinning is the single-needle configuration, there are other configurations designed to modify the morphology of the membrane for specific applications, such as coaxial electrospinning, which allows the production of nanofibers with a core–shell structure [16,17]. Advances in engineering have led to scalable electrospinning configurations, including needleless and centrifugally assisted electrospinning techniques [18,19].

Another essential factor influencing electrospinning is the shape of the fiber collector. Among the most common and easy-to-implement designs are static flat-plate and rotating drum collectors, the latter being the most widely used [20]. However, rotating drum collectors can lead to uneven fiber accumulation at different points on the collector, which affects membrane uniformity [21]. Although various mechanisms have been developed to reduce fiber accumulation and produce more uniform membranes, most rely on rotating drum collectors instead of flat-plate collectors. Additionally, these are typically implemented in professional-grade equipment, which comes at a high monetary cost [[22], [23], [24]].

Innovation in the design of electrospinning equipment is an area of ongoing development, with the primary goals of improving process efficiency, fiber reproducibility, and scalability. Some researchers have developed low-cost approaches to address the challenges associated with the electrospinning technique. For example, equipment using homemade voltage sources capable of reaching up to 9 kV and syringe pumps created with 3D printing have been evaluated for fabricating polylactic acid fibers with average diameters of 523 nm [24]. These efforts were enhanced by increasing the voltage output of the high-voltage source to 36 kV and incorporating a rotating drum collector, achieving fiber diameters averaging 483 nm [22]. Other studies, such as that of Switz *et al*., focused on developing electrospinning devices with different types of collectors, including rotating drums and spinneret tips, for producing polycaprolactone fibers [25]. Wijayanti *et al*. addressed the design of an electrospinning system featuring a custom-built syringe pump and a safety control system managed by Arduino to produce polyvinyl alcohol fibers [23].

Few studies verify and control the thickness consistency of the nanofibrous membranes produced. Within this context, the present article addresses the development of a novel electrospinning device designed to overcome two key challenges in the production of nanofibrous membranes: achieving membrane uniformity and ensuring economic accessibility. This innovative design features a programmable bidirectional collector mounted on a robust structural frame, which can move along the X and Y axes using accessible components, such as stepper motors controlled by open-source software (Universal G-code Sender, UGS). This approach enables more uniform fiber deposition, significantly improving the thickness reproducibility of the fabricated membranes. Furthermore, the use of widely available materials and the integration of digital technologies simplify the scalability of the equipment for various applications. Tests conducted with the system demonstrated its ability to produce uniform membranes with fibers in the nanometer and submicrometer range. The uniformity and quality of the resulting membranes suggest that this equipment can replace high-cost commercial systems in low-budget laboratories or laboratories that are just starting up their work, offering an affordable and efficient alternative for producing nanofibrous membranes.

## 2 Hardware description

**Fig. 1:**
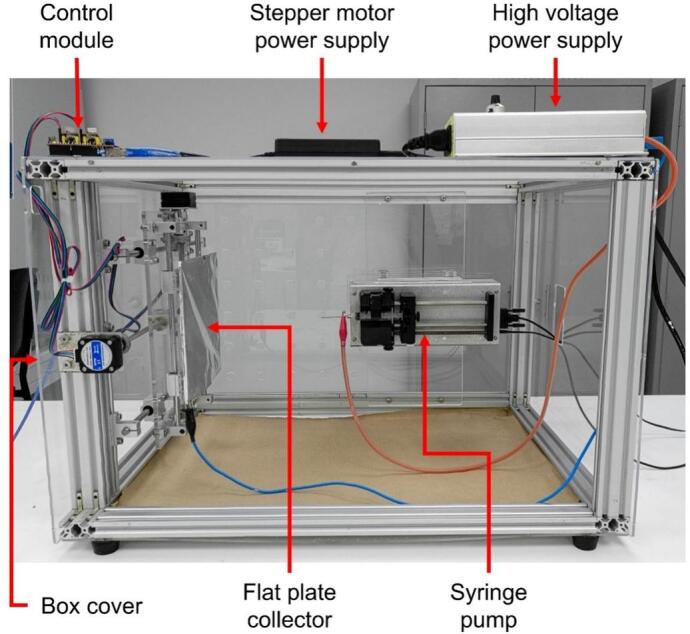
Main parts of the electrospinning equipment.

The developed equipment is an electrospinning system designed to optimize the production of uniform nanofibrous membranes through an innovative and cost-effective approach. This model integrates five main components: a bidirectional collector, a syringe pump, a high-voltage power supply, a control module, and a protective enclosure. Below is a detailed description of each component, highlighting the customizations and advantages over previously reported systems. Some critical components, such as the syringe pump and high-voltage power supply, were sourced from reputable suppliers but in their low-cost versions to ensure acceptable reproducibility. However, these components can be replaced with cheaper alternatives or homemade versions. The system, along with all its components, is illustrated in Fig. 1.

### 2.1 Bidirectional flat plate collector

**Fig. 2:**
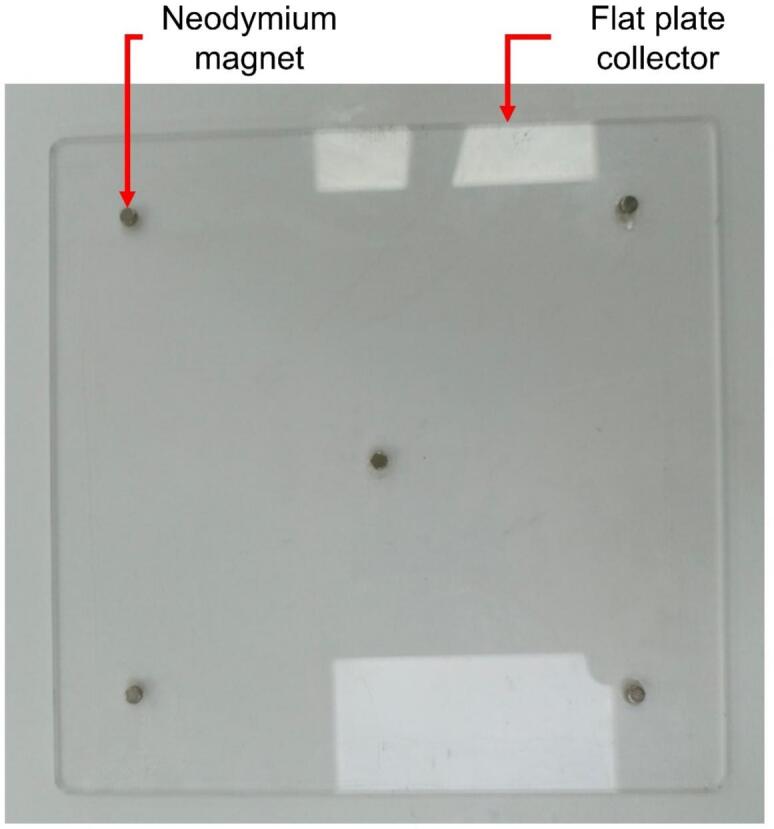
Collector flat plate.

The core of the innovative design lies in the collector, a flat acrylic (polymethyl methacrylate) plate measuring 200 mm × 180 mm × 3 mm, coated with aluminum foil to ensure electrical conductivity. This collector is mounted on a programmable bidirectional system that allows independent movement along the X and Y axes. The motion is achieved using two linear motion guides positioned at 90° angles, driven by two NEMA 17 stepper motors (model 17HE08-1004S). These guides are fixed together with an acrylic plate measuring 330 mm × 200 mm × 5 mm, onto which the collector plate covered in aluminum foil is mounted using five N35 neodymium magnets (Fig. 2). The precision of these motors, controlled by an Arduino Uno module and a CNC Shield with two A4988 drivers, enables customizable trajectories using the open-source software Universal G-code Sender (UGS). This design ensures uniform fiber deposition on the collector, resulting in more homogeneous membranes compared to conventional static or rotating collectors. Moreover, the bidirectional movement improves thickness reproducibility and membrane uniformity. The structure of the collector and its linear motion guide assembly is shown in Fig. 2.

### 2.2 High-voltage power supply

**Fig. 3:**
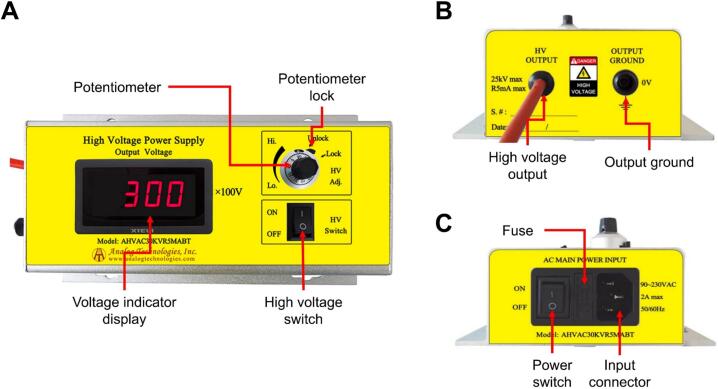
High voltage power supply: A) Front view. B) Left side view. C) Right side view.

The equipment employs a high-voltage power supply (model AHVAC30KVR5MABT from Analog Technologies), capable of generating voltages of up to 30 kV with a current of 0.5 mA. This model was chosen for its reliability, high precision, and ease of use, all essential characteristics for a safe and efficient electrospinning system. Its compact design allows manual adjustment via a rotary knob, and the inclusion of a voltage lock ensures operational stability. This component guarantees the generation of the electrostatic field required to stretch and deposit nanometric fibers. The high-voltage power supply is depicted in Fig. 3.

### 2.3 Syringe pump

**Fig. 4:**
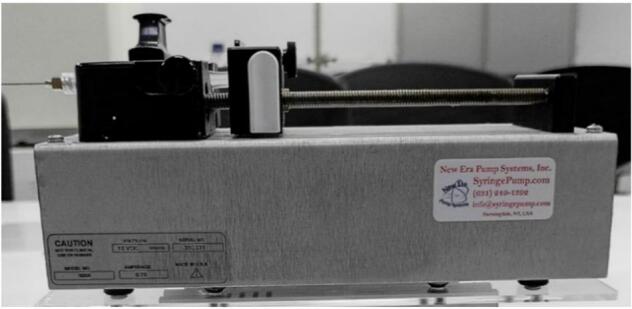
Syringe pump.

The injection of the polymer solution is managed by a SyringePump NE-500 from New Era Pump System Inc., mounted on a custom-designed acrylic base. This base allows for easy adjustment of the distance between the needle and the collector, adapting the equipment to different experimental conditions. The pump is programmable, with adjustable flow rates ranging from 0.73 µL/h to 2100 mL/h, providing versatility to work with various materials and configurations. Operation is initiated and stopped via a foot pedal that serves as an external actuator. The syringe pump assembly is shown in Fig. 4.

### 2.4 Protective enclosure

To ensure user safety, the equipment is enclosed in an acrylic box measuring 686 mm × 469 mm × 446 mm, constructed from extruded aluminum profiles (20 mm × 40 mm) and transparent acrylic panels. This enclosure not only provides structural support for the collector and syringe pump but also acts as an insulator between the equipment’s interior and the operator. One of the side panels is hinged for easy access during adjustments and cleaning, while rubber feet enhance stability and reduce the risk of electric shock.

### 2.5 Control module

The control system combines automation and manual operation. The collector is programmed using Universal G-code Sender (UGS) software, while the syringe pump and high-voltage power supply are manually operated. This hybrid approach allows users to adjust critical parameters in real time while maintaining high precision in the collector’s movements. Key components of the control module include a laptop and a 12 V power supply adapted from a laptop charger. The main elements are described as follows:

● Arduino UNO: A microcontroller board based on the ATmega328P, featuring 14 digital input/output pins, six analog inputs, a USB connection, a power jack, ICSP headers, and a reset button [26].
● CNC Shield: An attachable board for the Arduino Uno, allowing control of up to four stepper motors. It includes four sockets for A4988 or DVR8825 drivers, supporting end-stops for each axis, and is compatible with GRBL firmware [27].
● A4988 Driver: A controller for bipolar stepper motors, capable of delivering a maximum current of 2 A. It supports voltages ranging from 8 V to 35 V and includes an adjustable current input potentiometer [28].

**Fig. 5:**
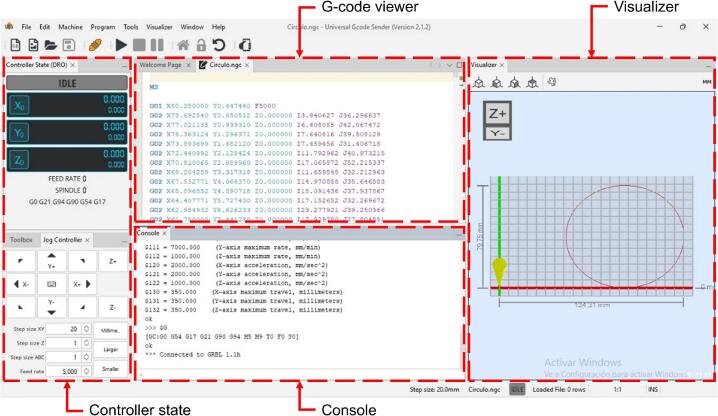
Graphic interface of the UGS program.

**Fig. 6:**
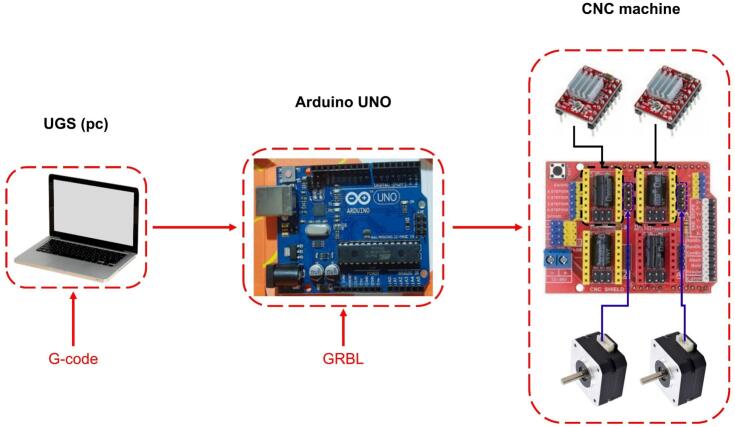
Operation of the control module.

The UGS software is an open-source program used to send G-code commands, the working language of CNC machines. Its graphical interface allows users to create or upload G-code, execute it, and visualize the motion pattern in real time. The graphical interface of the UGS software [29] is shown in Fig. 5, while the operation of the control module is illustrated in Fig. 6.

For this case, the collector was programmed with a G-code that enables the motors and the collector plate to describe a circular motion lasting approximately three hours. The type of motion and its duration can be modified in the G-code.

In addition to the technical details, this setup offers features that can help researchers in their work across different applications:

● **Membrane Reproducibility:** Thanks to the bidirectional collector, electrospinning can produce membranes with consistent thickness and fiber distribution compared to static methods, making membrane fabrication more reproducible for water treatment and tissue engineering.
● **Adaptable to Diverse Materials:** The programmable syringe pump, adjustable collector distance, and movement enable the use of various solutions, such as collagen-hyaluronic acid for scaffolds and polymer-nanoparticle composites (like polystyrene-silver) for antibacterial membranes, broadening its applications beyond traditional electrospinning tasks.
● **Low-Cost and Customizable Setup:** This equipment offers an affordable option for labs with limited budgets because it uses inexpensive components and open-source control software.

## 3 Design files

A summary of all the files used for designing this equipment is shown in Table 1.

**Table 1:** Design file summary.

Design file name	File type	Open-source license	Location of the file
Cover box structure	Blender CAD file	Creative Commons Attribution (CC BY) 4.0 license	https://doi.org/10.17632/44t3m6yzt9.2
Linear motion guide kit assembly 400 mm	Blender CAD file	Creative Commons Attribution (CC BY) 4.0 license	https://doi.org/10.17632/44t3m6yzt9.2
Linear motion guide kit assembly 300 mm	Blender CAD file	Creative Commons Attribution (CC BY) 4.0 license	https://doi.org/10.17632/44t3m6yzt9.2
Collector flat plate assembly	Blender CAD file	Creative Commons Attribution (CC BY) 4.0 license	https://doi.org/10.17632/44t3m6yzt9.2
Syringe pump structure bracket	Blender CAD file	Creative Commons Attribution (CC BY) 4.0 license	https://doi.org/10.17632/44t3m6yzt9.2
Pump assembly	Blender CAD file	Creative Commons Attribution (CC BY) 4.0 license	https://doi.org/10.17632/44t3m6yzt9.2
Finished Electrospinning	Blender CAD file	Creative Commons Attribution (CC BY) 4.0 license	https://doi.org/10.17632/44t3m6yzt9.2
Acrylic plate 1800 × 1200 × 3 mm	Vectorized PDF file	Creative Commons Attribution (CC BY) 4.0 license	https://doi.org/10.17632/44t3m6yzt9.2
Acrylic plate 330 × 200 × 5 mm	Vectorized PDF file	Creative Commons Attribution (CC BY) 4.0 license	https://doi.org/10.17632/44t3m6yzt9.2
Acrylic plate 120 × 250 × 3 mm	Vectorized PDF file	Creative Commons Attribution (CC BY) 4.0 license	https://doi.org/10.17632/44t3m6yzt9.2
Acrylic plate 130 × 160 × 3 mm	Vectorized PDF file	Creative Commons Attribution (CC BY) 4.0 license	https://doi.org/10.17632/44t3m6yzt9.2
Acrylic plate 180 × 400 × 5 mm	Vectorized PDF file	Creative Commons Attribution (CC BY) 4.0 license	https://doi.org/10.17632/44t3m6yzt9.2
Circle	UGS G-code file	Creative Commons Attribution (CC BY) 4.0 license	https://doi.org/10.17632/44t3m6yzt9.2
G-code_Movement	Python file	Creative Commons Attribution (CC BY) 4.0 license	https://doi.org/10.17632/44t3m6yzt9.2

To control the movement of the bidirectional collector, a G-code trajectory derived from a circular pattern previously designed in Inkscape was used. The circular pattern was exported in.ngc format, which is the mother language of UGS, using the built-in G-code export function provided by Inkscape. Then, this.ngc file was processed using a custom Python script, which enabled control over the duration of the electrospinning process by repeating the trajectory a defined number of times through a loop command. Below is a detailed description of the code sections and their functions:

● file = open(“Circle.ngc”, “w”)

This command creates a.ngc file where the circular G-code trajectory will be written.

● file.write(“”“\nM3 \n
  G01 X80.250000 Y0.447440 F5000
  G02 X78.692540 Y0.650512 Z0.000000 I3.940627J36.296637
  … (142 more command lines…)
  G02 X80.250000 Y0.447440 Z0.000000 I1.072932J24.412419 \n ”“”)

This line uses M3 command to activate the spindle. G01 line moves the collector to its starting position (X80.250000 Y0.447440) at a controlled feed rate (F5000). Then, G02 line starts moving the collector making a clockwise arc (X78.692540 Y0.650512 Z0.000000), with specific arc centers to the starting point (I3.940627J36.296637). This process continues over 142 more command lines until reaching the final command line (G02 X80.250000 Y0.447440 Z0.000000 I1.072932J24.412419 \n “”“) to return to the initial position, which defines one complete circular trajectory.

● for i in range(4199):
  file.write(“”“…”“”)

This loop generates the previous circular trajectory 4199 times into the.ngc file. Using this command, it is possible to directly determine the duration of the electrospinning process and the total length of collector movement.

● file.write(“\nM5 G01 X0 Y0 \n \nM2″)

This is the final line of the code. M5 is used to stop the spindle. G01 X0 Y0 line moves the collector to the origin and M2 command ends the G-code program. The complete G-code is provided below:

“”“

@author: Jhonatan A. Gutierrez-Rivera, Andres F. Roca-Arroyo, David A. Castilla-Casadiego, and Alberto Albis

“”“

file = open(“Circle.ngc”,“w”) #Open the.ngc file to write the Gcode. Then, it is read by the UGS software

file.write(“”“\nM3 \n

G01 X80.250000 Y0.447440 F5000

G02 X78.692540 Y0.650512 Z0.000000 I3.940627 J36.296637

G02 X77.021135 Y0.939310 Z0.000000 I6.408085 J42.067472

G02 X75.363124 Y1.296371 Z0.000000 I7.640816 J39.508129

G02 X73.893699 Y1.682120 Z0.000000 I7.459456 J31.406718

G02 X72.440992 Y2.128424 Z0.000000 I11.792962 J40.973215

G02 X70.810065 Y2.689960 Z0.000000 I17.065872 J52.215337

G02 X69.204259 Y3.317318 Z0.000000 I11.659548 J32.212963

G02 X67.532771 Y4.066370 Z0.000000 I14.970888 J35.646583

G02 X65.896852 Y4.890718 Z0.000000 I18.091436 J37.937867

G02 X64.407771 Y5.727430 Z0.000000 I17.152652 J32.269672

G02 X62.954932 Y6.626233 Z0.000000 I23.277921 J39.250366

G02 X61.750000 Y7.441280 Z0.000000 I17.929780 J27.804851

G02 X60.579248 Y8.305112 Z0.000000 I22.190927 J31.300641

G02 X59.250000 Y9.360150 Z0.000000 I31.781155 J41.406141

G02 X57.967082 Y10.469805 Z0.000000 I20.729063 J25.262167

G02 X56.559385 Y11.812310 Z0.000000 I28.947599 J31.762577

G02 X55.208267 Y13.212315 Z0.000000 I36.501579 J36.578939

G02 X54.211127 Y14.339060 Z0.000000 I22.631187 J21.032611

G02 X53.266036 Y15.510093 Z0.000000 I27.313265 J23.010270

G02 X52.234301 Y16.896930 Z0.000000 I37.281472 J28.812677

G02 X51.257046 Y18.322454 Z0.000000 I34.586587 J24.758249

G02 X50.429380 Y19.648000 Z0.000000 I29.074073 J19.074917

G02 X49.652433 Y21.004408 Z0.000000 I43.321599 J25.715207

G02 X49.075983 Y22.098160 Z0.000000 I27.584377 J15.236836

G02 X48.539471 Y23.212195 Z0.000000 I33.243881 J16.696252

G02 X47.964359 Y24.508020 Z0.000000 I44.415477 J20.487968

G02 X47.441067 Y25.824953 Z0.000000 I26.095525 J11.131671

G02 X46.838134 Y27.562500 Z0.000000 I44.831340 J16.529955

G02 X46.290163 Y29.318777 Z0.000000 I71.005190 J23.117741

G02 X46.017150 Y30.312500 Z0.000000 I22.450471 J6.702358

G02 X45.782320 Y31.316123 Z0.000000 I30.701568 J7.712904

G02 X45.511913 Y32.642140 Z0.000000 I53.330588 J11.565984

G02 X45.290306 Y33.976554 Z0.000000 I27.452500 J5.244652

G02 X45.066448 Y35.794830 Z0.000000 I50.765376 J7.172939

G02 X44.929133 Y37.620722 Z0.000000 I30.798723 J3.234314

G02 X44.875000 Y40.000000 Z0.000000 I52.260908 J2.379278

G02 X44.928988 Y42.379305 Z0.000000 I52.456465 J0.000000

G02 X45.066714 Y44.222310 Z0.000000 I31.599517 J-1.434758

G02 X45.301667 Y46.055908 Z0.000000 I38.898410 J-4.052491

G02 X45.622795 Y47.875000 Z0.000000 I38.607152 J-5.877517

G02 X46.027924 Y49.677275 Z0.000000 I39.845792 J-8.010168

G02 X46.478009 Y51.312500 Z0.000000 I33.249159 J-8.272059

G02 X46.991819 Y52.929742 Z0.000000 I56.887844 J-17.183470

G02 X47.436820 Y54.187500 Z0.000000 I34.848409 J-11.621927

G02 X47.935408 Y55.424507 Z0.000000 I25.061020 J-9.382117

G02 X48.692628 Y57.092230 Z0.000000 I46.322931 J-20.026883

G02 X49.515607 Y58.728245 Z0.000000 I38.074713 J-18.128056

G02 X50.350612 Y60.217230 Z0.000000 I32.368723 J-17.173369

G02 X51.252103 Y61.667053 Z0.000000 I33.536669 J-19.847703

G02 X52.230867 Y63.098440 Z0.000000 I33.671587 J-21.973881

G02 X53.266262 Y64.489711 Z0.000000 I38.215770 J-27.359578

G02 X54.212404 Y65.660940 Z0.000000 I28.019334 J-21.666797

G02 X55.209763 Y66.788669 Z0.000000 I24.611591 J-20.761503

G02 X56.498162 Y68.126840 Z0.000000 I35.959474 J-33.332664

G02 X57.836328 Y69.415230 Z0.000000 I34.667653 J-34.667892

G02 X58.964062 Y70.412600 Z0.000000 I21.895463 J-23.621110

G02 X60.135276 Y71.358751 Z0.000000 I22.850624 J-27.088177

G02 X61.526562 Y72.394230 Z0.000000 I28.769819 J-37.203160

G02 X62.955557 Y73.376874 Z0.000000 I26.584499 J-37.129735

G02 X64.187500 Y74.150540 Z0.000000 I17.699117 J-26.815422

G02 X65.454907 Y74.864416 Z0.000000 I14.821843 J-24.832502

G02 X67.312500 Y75.808610 Z0.000000 I27.817117 J-52.427601

G02 X69.204128 Y76.681644 Z0.000000 I22.442001 J-46.139902

G02 X70.562500 Y77.229260 Z0.000000 I10.157054 J-23.236217

G02 X71.944986 Y77.715563 Z0.000000 I15.813748 J-42.747879

G02 X73.431804 Y78.187000 Z0.000000 I16.338372 J-48.947636

G02 X74.935741 Y78.597569 Z0.000000 I8.817672 J-29.339856

G02 X76.744304 Y79.001330 Z0.000000 I10.332939 J-42.031789

G02 X78.569080 Y79.323327 Z0.000000 I7.738941 J-38.525464

G02 X80.402695 Y79.558290 Z0.000000 I5.886895 J-38.668334

G02 X82.245696 Y79.696012 Z0.000000 I3.277840 J-31.463662

G02 X84.625000 Y79.750000 Z0.000000 I2.379304 J-52.402586

G02 X87.004265 Y79.695673 Z0.000000 I0.000000 J-52.127194

G02 X88.841696 Y79.557340 Z0.000000 I-1.426429 J-31.218990

G02 X90.670616 Y79.326837 Z0.000000 I-4.641124 J-44.195884

G02 X92.293210 Y79.049410 Z0.000000 I-5.159829 J-35.062182

G02 X93.901899 Y78.699911 Z0.000000 I-7.373197 J-37.814680

G02 X95.423820 Y78.298890 Z0.000000 I-8.212275 J-34.254983

G02 X96.930353 Y77.841514 Z0.000000 I-14.729851 J-51.227982

G02 X98.187500 Y77.415070 Z0.000000 I-11.543760 J-36.096936

G02 X99.429461 Y76.946326 Z0.000000 I-13.719567 J-38.230391

G02 X100.514160 Y76.493230 Z0.000000 I-11.735037 J-29.618254

G02 X101.584422 Y76.006402 Z0.000000 I-18.545454 J-42.190927

G02 X102.613100 Y75.505990 Z0.000000 I-18.574473 J-39.490356

G02 X103.624305 Y74.971767 Z0.000000 I-12.750515 J-25.358956

G02 X104.978570 Y74.194690 Z0.000000 I-25.661115 J-46.290023

G02 X106.302560 Y73.367974 Z0.000000 I-17.719024 J-29.850649

G02 X107.728070 Y72.390700 Z0.000000 I-23.345334 J-35.581206

G02 X109.115049 Y71.359126 Z0.000000 I-27.618674 J-38.582243

G02 X110.285940 Y70.414900 Z0.000000 I-21.965652 J-28.436695

G02 X111.411564 Y69.417576 Z0.000000 I-19.023666 J-22.604835

G02 X112.880360 Y67.995570 Z0.000000 I-37.235445 J-39.930204

G02 X114.283297 Y66.509636 Z0.000000 I-27.674969 J-27.534447

G02 X115.574990 Y64.987540 Z0.000000 I-27.741370 J-24.851219

G02 X116.792818 Y63.404903 Z0.000000 I-37.007803 J-29.737117

G02 X117.778260 Y61.996110 Z0.000000 I-28.321909 J-20.860051

G02 X118.701829 Y60.545535 Z0.000000 I-37.729518 J-25.041360

G02 X119.481770 Y59.204960 Z0.000000 I-31.452464 J-19.196094

G02 X120.203770 Y57.832374 Z0.000000 I-31.359506 J-17.371734

G02 X120.934660 Y56.291620 Z0.000000 I-38.786565 J-19.342982

G02 X121.588485 Y54.717471 Z0.000000 I-26.263285 J-11.831354

G02 X122.370320 Y52.524160 Z0.000000 I-50.237767 J-19.143921

G02 X123.052826 Y50.298188 Z0.000000 I-47.523162 J-15.788702

G02 X123.484930 Y48.562500 Z0.000000 I-28.446636 J-8.003500

G02 X123.834602 Y46.807103 Z0.000000 I-51.118808 J-11.095304

G02 X124.086050 Y45.250000 Z0.000000 I-39.974636 J-7.254137

G02 X124.229775 Y43.682037 Z0.000000 I-15.822942 J-2.240959

G02 X124.307340 Y40.000000 Z0.000000 I-87.355442 J-3.682037

G02 X124.229766 Y36.317962 Z0.000000 I-87.422875 J0.000000

G02 X124.086050 Y34.750000 Z0.000000 I-15.970059 J0.673217

G02 X123.834603 Y33.192906 Z0.000000 I-40.228503 J5.697442

G02 X123.484930 Y31.437500 Z0.000000 I-51.467314 J9.339645

G02 X123.052754 Y29.701835 Z0.000000 I-28.838440 J6.259065

G02 X122.368000 Y27.468900 Z0.000000 I-48.435595 J13.631835

G02 X121.583928 Y25.268665 Z0.000000 I-51.407100 J17.079528

G02 X120.946460 Y23.736360 Z0.000000 I-25.555056 J9.732635

G02 X120.232786 Y22.237094 Z0.000000 I-38.437222 J17.377210

G02 X119.492130 Y20.829960 Z0.000000 I-34.599317 J17.313143

G02 X118.694662 Y19.454196 Z0.000000 I-34.351919 J18.993245

G02 X117.783020 Y18.014490 Z0.000000 I-38.535300 J23.392601

G02 X116.809100 Y16.616780 Z0.000000 I-28.233827 J18.635083

G02 X115.578380 Y15.014490 Z0.000000 I-38.345386 J28.179304

G02 X114.270022 Y13.475983 Z0.000000 I-27.635692 J22.176039

G02 X112.750230 Y11.874770 Z0.000000 I-31.448294 J28.327289

G02 X111.149017 Y10.354978 Z0.000000 I-29.928503 J29.928503

G02 X109.610510 Y9.046620 Z0.000000 I-23.714545 J26.327334

G02 X108.008240 Y7.815875 Z0.000000 I-29.804072 J37.142679

G02 X106.606680 Y6.839010 Z0.000000 I-20.165663 J27.438868

G02 X105.163804 Y5.923259 Z0.000000 I-26.044832 J39.442188

G02 X103.826130 Y5.144020 Z0.000000 I-20.179447 J33.103096

G02 X102.457009 Y4.421962 Z0.000000 I-16.945385 J30.471735

G02 X100.954960 Y3.710330 Z0.000000 I-17.962269 J35.972123

G02 X99.426474 Y3.056921 Z0.000000 I-19.115013 J42.600309

G02 X98.187500 Y2.585100 Z0.000000 I-11.189749 J27.520969

G02 X96.931878 Y2.158077 Z0.000000 I-16.195304 J45.561305

G02 X95.653190 Y1.761800 Z0.000000 I-15.228185 J46.876418

G02 X94.360768 Y1.415428 Z0.000000 I-7.549986 J25.586928

G02 X92.528190 Y1.007750 Z0.000000 I-12.320627 J51.060529

G02 X90.680611 Y0.677547 Z0.000000 I-7.753199 J38.047386

G02 X88.847305 Y0.441330 Z0.000000 I-5.728052 J37.223666

G02 X87.004219 Y0.308806 Z0.000000 I-3.003370 J28.886773

G02 X84.409805 Y0.263910 Z0.000000 I-2.287718 J57.216982

G02 X81.815357 Y0.328164 Z0.000000 I0.379277 J67.725428

G02 X80.250000 Y0.447440 Z0.000000 I1.072932 J24.412419 \n “”“)

for i in range(4199): #Change the loop duration

print(i, file.write(

“”“

G01 X80.250000 Y0.447440 F5000

G02 X78.692540 Y0.650512 Z0.000000 I3.940627 J36.296637

G02 X77.021135 Y0.939310 Z0.000000 I6.408085 J42.067472

G02 X75.363124 Y1.296371 Z0.000000 I7.640816 J39.508129

G02 X73.893699 Y1.682120 Z0.000000 I7.459456 J31.406718

G02 X72.440992 Y2.128424 Z0.000000 I11.792962 J40.973215

G02 X70.810065 Y2.689960 Z0.000000 I17.065872 J52.215337

G02 X69.204259 Y3.317318 Z0.000000 I11.659548 J32.212963

G02 X67.532771 Y4.066370 Z0.000000 I14.970888 J35.646583

G02 X65.896852 Y4.890718 Z0.000000 I18.091436 J37.937867

G02 X64.407771 Y5.727430 Z0.000000 I17.152652 J32.269672

G02 X62.954932 Y6.626233 Z0.000000 I23.277921 J39.250366

G02 X61.750000 Y7.441280 Z0.000000 I17.929780 J27.804851

G02 X60.579248 Y8.305112 Z0.000000 I22.190927 J31.300641

G02 X59.250000 Y9.360150 Z0.000000 I31.781155 J41.406141

G02 X57.967082 Y10.469805 Z0.000000 I20.729063 J25.262167

G02 X56.559385 Y11.812310 Z0.000000 I28.947599 J31.762577

G02 X55.208267 Y13.212315 Z0.000000 I36.501579 J36.578939

G02 X54.211127 Y14.339060 Z0.000000 I22.631187 J21.032611

G02 X53.266036 Y15.510093 Z0.000000 I27.313265 J23.010270

G02 X52.234301 Y16.896930 Z0.000000 I37.281472 J28.812677

G02 X51.257046 Y18.322454 Z0.000000 I34.586587 J24.758249

G02 X50.429380 Y19.648000 Z0.000000 I29.074073 J19.074917

G02 X49.652433 Y21.004408 Z0.000000 I43.321599 J25.715207

G02 X49.075983 Y22.098160 Z0.000000 I27.584377 J15.236836

G02 X48.539471 Y23.212195 Z0.000000 I33.243881 J16.696252

G02 X47.964359 Y24.508020 Z0.000000 I44.415477 J20.487968

G02 X47.441067 Y25.824953 Z0.000000 I26.095525 J11.131671

G02 X46.838134 Y27.562500 Z0.000000 I44.831340 J16.529955

G02 X46.290163 Y29.318777 Z0.000000 I71.005190 J23.117741

G02 X46.017150 Y30.312500 Z0.000000 I22.450471 J6.702358

G02 X45.782320 Y31.316123 Z0.000000 I30.701568 J7.712904

G02 X45.511913 Y32.642140 Z0.000000 I53.330588 J11.565984

G02 X45.290306 Y33.976554 Z0.000000 I27.452500 J5.244652

G02 X45.066448 Y35.794830 Z0.000000 I50.765376 J7.172939

G02 X44.929133 Y37.620722 Z0.000000 I30.798723 J3.234314

G02 X44.875000 Y40.000000 Z0.000000 I52.260908 J2.379278

G02 X44.928988 Y42.379305 Z0.000000 I52.456465 J0.000000

G02 X45.066714 Y44.222310 Z0.000000 I31.599517 J-1.434758

G02 X45.301667 Y46.055908 Z0.000000 I38.898410 J-4.052491

G02 X45.622795 Y47.875000 Z0.000000 I38.607152 J-5.877517

G02 X46.027924 Y49.677275 Z0.000000 I39.845792 J-8.010168

G02 X46.478009 Y51.312500 Z0.000000 I33.249159 J-8.272059

G02 X46.991819 Y52.929742 Z0.000000 I56.887844 J-17.183470

G02 X47.436820 Y54.187500 Z0.000000 I34.848409 J-11.621927

G02 X47.935408 Y55.424507 Z0.000000 I25.061020 J-9.382117

G02 X48.692628 Y57.092230 Z0.000000 I46.322931 J-20.026883

G02 X49.515607 Y58.728245 Z0.000000 I38.074713 J-18.128056

G02 X50.350612 Y60.217230 Z0.000000 I32.368723 J-17.173369

G02 X51.252103 Y61.667053 Z0.000000 I33.536669 J-19.847703

G02 X52.230867 Y63.098440 Z0.000000 I33.671587 J-21.973881

G02 X53.266262 Y64.489711 Z0.000000 I38.215770 J-27.359578

G02 X54.212404 Y65.660940 Z0.000000 I28.019334 J-21.666797

G02 X55.209763 Y66.788669 Z0.000000 I24.611591 J-20.761503

G02 X56.498162 Y68.126840 Z0.000000 I35.959474 J-33.332664

G02 X57.836328 Y69.415230 Z0.000000 I34.667653 J-34.667892

G02 X58.964062 Y70.412600 Z0.000000 I21.895463 J-23.621110

G02 X60.135276 Y71.358751 Z0.000000 I22.850624 J-27.088177

G02 X61.526562 Y72.394230 Z0.000000 I28.769819 J-37.203160

G02 X62.955557 Y73.376874 Z0.000000 I26.584499 J-37.129735

G02 X64.187500 Y74.150540 Z0.000000 I17.699117 J-26.815422

G02 X65.454907 Y74.864416 Z0.000000 I14.821843 J-24.832502

G02 X67.312500 Y75.808610 Z0.000000 I27.817117 J-52.427601

G02 X69.204128 Y76.681644 Z0.000000 I22.442001 J-46.139902

G02 X70.562500 Y77.229260 Z0.000000 I10.157054 J-23.236217

G02 X71.944986 Y77.715563 Z0.000000 I15.813748 J-42.747879

G02 X73.431804 Y78.187000 Z0.000000 I16.338372 J-48.947636

G02 X74.935741 Y78.597569 Z0.000000 I8.817672 J-29.339856

G02 X76.744304 Y79.001330 Z0.000000 I10.332939 J-42.031789

G02 X78.569080 Y79.323327 Z0.000000 I7.738941 J-38.525464

G02 X80.402695 Y79.558290 Z0.000000 I5.886895 J-38.668334

G02 X82.245696 Y79.696012 Z0.000000 I3.277840 J-31.463662

G02 X84.625000 Y79.750000 Z0.000000 I2.379304 J-52.402586

G02 X87.004265 Y79.695673 Z0.000000 I0.000000 J-52.127194

G02 X88.841696 Y79.557340 Z0.000000 I-1.426429 J-31.218990

G02 X90.670616 Y79.326837 Z0.000000 I-4.641124 J-44.195884

G02 X92.293210 Y79.049410 Z0.000000 I-5.159829 J-35.062182

G02 X93.901899 Y78.699911 Z0.000000 I-7.373197 J-37.814680

G02 X95.423820 Y78.298890 Z0.000000 I-8.212275 J-34.254983

G02 X96.930353 Y77.841514 Z0.000000 I-14.729851 J-51.227982

G02 X98.187500 Y77.415070 Z0.000000 I-11.543760 J-36.096936

G02 X99.429461 Y76.946326 Z0.000000 I-13.719567 J-38.230391

G02 X100.514160 Y76.493230 Z0.000000 I-11.735037 J-29.618254

G02 X101.584422 Y76.006402 Z0.000000 I-18.545454 J-42.190927

G02 X102.613100 Y75.505990 Z0.000000 I-18.574473 J-39.490356

G02 X103.624305 Y74.971767 Z0.000000 I-12.750515 J-25.358956

G02 X104.978570 Y74.194690 Z0.000000 I-25.661115 J-46.290023

G02 X106.302560 Y73.367974 Z0.000000 I-17.719024 J-29.850649

G02 X107.728070 Y72.390700 Z0.000000 I-23.345334 J-35.581206

G02 X109.115049 Y71.359126 Z0.000000 I-27.618674 J-38.582243

G02 X110.285940 Y70.414900 Z0.000000 I-21.965652 J-28.436695

G02 X111.411564 Y69.417576 Z0.000000 I-19.023666 J-22.604835

G02 X112.880360 Y67.995570 Z0.000000 I-37.235445 J-39.930204

G02 X114.283297 Y66.509636 Z0.000000 I-27.674969 J-27.534447

G02 X115.574990 Y64.987540 Z0.000000 I-27.741370 J-24.851219

G02 X116.792818 Y63.404903 Z0.000000 I-37.007803 J-29.737117

G02 X117.778260 Y61.996110 Z0.000000 I-28.321909 J-20.860051

G02 X118.701829 Y60.545535 Z0.000000 I-37.729518 J-25.041360

G02 X119.481770 Y59.204960 Z0.000000 I-31.452464 J-19.196094

G02 X120.203770 Y57.832374 Z0.000000 I-31.359506 J-17.371734

G02 X120.934660 Y56.291620 Z0.000000 I-38.786565 J-19.342982

G02 X121.588485 Y54.717471 Z0.000000 I-26.263285 J-11.831354

G02 X122.370320 Y52.524160 Z0.000000 I-50.237767 J-19.143921

G02 X123.052826 Y50.298188 Z0.000000 I-47.523162 J-15.788702

G02 X123.484930 Y48.562500 Z0.000000 I-28.446636 J-8.003500

G02 X123.834602 Y46.807103 Z0.000000 I-51.118808 J-11.095304

G02 X124.086050 Y45.250000 Z0.000000 I-39.974636 J-7.254137

G02 X124.229775 Y43.682037 Z0.000000 I-15.822942 J-2.240959

G02 X124.307340 Y40.000000 Z0.000000 I-87.355442 J-3.682037

G02 X124.229766 Y36.317962 Z0.000000 I-87.422875 J0.000000

G02 X124.086050 Y34.750000 Z0.000000 I-15.970059 J0.673217

G02 X123.834603 Y33.192906 Z0.000000 I-40.228503 J5.697442

G02 X123.484930 Y31.437500 Z0.000000 I-51.467314 J9.339645

G02 X123.052754 Y29.701835 Z0.000000 I-28.838440 J6.259065

G02 X122.368000 Y27.468900 Z0.000000 I-48.435595 J13.631835

G02 X121.583928 Y25.268665 Z0.000000 I-51.407100 J17.079528

G02 X120.946460 Y23.736360 Z0.000000 I-25.555056 J9.732635

G02 X120.232786 Y22.237094 Z0.000000 I-38.437222 J17.377210

G02 X119.492130 Y20.829960 Z0.000000 I-34.599317 J17.313143

G02 X118.694662 Y19.454196 Z0.000000 I-34.351919 J18.993245

G02 X117.783020 Y18.014490 Z0.000000 I-38.535300 J23.392601

G02 X116.809100 Y16.616780 Z0.000000 I-28.233827 J18.635083

G02 X115.578380 Y15.014490 Z0.000000 I-38.345386 J28.179304

G02 X114.270022 Y13.475983 Z0.000000 I-27.635692 J22.176039

G02 X112.750230 Y11.874770 Z0.000000 I-31.448294 J28.327289

G02 X111.149017 Y10.354978 Z0.000000 I-29.928503 J29.928503

G02 X109.610510 Y9.046620 Z0.000000 I-23.714545 J26.327334

G02 X108.008240 Y7.815875 Z0.000000 I-29.804072 J37.142679

G02 X106.606680 Y6.839010 Z0.000000 I-20.165663 J27.438868

G02 X105.163804 Y5.923259 Z0.000000 I-26.044832 J39.442188

G02 X103.826130 Y5.144020 Z0.000000 I-20.179447 J33.103096

G02 X102.457009 Y4.421962 Z0.000000 I-16.945385 J30.471735

G02 X100.954960 Y3.710330 Z0.000000 I-17.962269 J35.972123

G02 X99.426474 Y3.056921 Z0.000000 I-19.115013 J42.600309

G02 X98.187500 Y2.585100 Z0.000000 I-11.189749 J27.520969

G02 X96.931878 Y2.158077 Z0.000000 I-16.195304 J45.561305

G02 X95.653190 Y1.761800 Z0.000000 I-15.228185 J46.876418

G02 X94.360768 Y1.415428 Z0.000000 I-7.549986 J25.586928

G02 X92.528190 Y1.007750 Z0.000000 I-12.320627 J51.060529

G02 X90.680611 Y0.677547 Z0.000000 I-7.753199 J38.047386

G02 X88.847305 Y0.441330 Z0.000000 I-5.728052 J37.223666

G02 X87.004219 Y0.308806 Z0.000000 I-3.003370 J28.886773

G02 X84.409805 Y0.263910 Z0.000000 I-2.287718 J57.216982

G02 X81.815357 Y0.328164 Z0.000000 I0.379277 J67.725428

G02 X80.250000 Y0.447440 Z0.000000 I1.072932 J24.412419 \n “”“))

file.write(“\nM5 G01 X0 Y0 \n \nM2″)

## 4 Bill of materials

See Table 2.

**Table 2:** Bill of materials summary.

Designator	Component	Number	Cost per unit-USD	Total cost-USD	Source of material	Material type
Box cover − Front/Back cover	Acrylic plate 680 × 443 × 3 mm	2	12	24	Local retailer	Acrylic
Box cover − Top/Button cover	Acrylic plate 680 × 460 × 3 mm	2	9	18	Local retailer	Acrylic
Box cover − Left/Right cover	Acrylic plate 446 × 443 × 3 mm	2	13	26	Local retailer	Acrylic
Box cover − Aluminum profile 600 × 40 × 20 mm	Aluminum profile 600 × 40 × 20 mm	4	9	36	Link 1	Aluminum
Box cover − Aluminum profile 460 × 40 × 20 mm	Aluminum profile 460 × 40 × 20 mm	4	6.9	27.6	Link 1	Aluminum
Box cover − Aluminum profile 400 × 40 × 20 mm	Aluminum profile 400 × 40 × 20 mm	5	6	30	Link 1	Aluminum
Box cover − 20 Set Silver 2020 Series T Slot L Shape Corner Bracket	20 Set Silver 2020 Series T Slot L Shape Corner Bracket	2	8	16	Link 2	Stainless steel
Box cover − Neodymium magnet	Neodymium magnet	6	0.25	1.5	Link 3	Neodymium
Box cover − Aluminum hinge	Aluminum hinge	2	0.3	0.6	Local retailer	Aluminum
Box cover − Rubber feet	Rubber feet	4	0.8	3.2	Local retailer	Rubber
Box cover − M5x12 screw	M5x12 screw bolts	23	0.07	1.61	Local retailer	Stainless steel
240 Pieces 2020 Series T Nuts, M3 M4 M5 T Slot Nut Assortment Kit	240 Pieces 2020 Series T Nuts, M3 M4 M5 T Slot Nut Assortment Kit	1	10	10	Link 4	Stainless steel
Flat collector − Linear motion guide kit 400 mm	Linear motion guide kit 400 mm	1	34	34	Link 5	Steel/Aluminum alloy
Flat collector − Linear motion guide kit 300 mm	Linear motion guide kit 300 mm	1	36	36	Link 5	Steel/Aluminum alloy
Flat collector − Linear motion guide coupling plate	Acrylic plate 330 × 200 × 5 mm	1	2	2	Local retailer	Acrylic
Flat collector − Collector bracket plate	Acrylic plate 200 × 180 × 3 mm	1	1	1	Local retailer	Acrylic
Flat collector − Flat collector plate	Acrylic plate 200 × 200 × 3 mm	1	2	2	Local retailer	Acrylic
Flat collector − Stepper motor	Stepper motor NEMA 17HE08-1004S	2	10	20	Link 6	Other
Flat collector − X-axis stepper motor bracket	NEMA 17HE08-1004S Straight bracket	1	3	3	Local retailer	Aluminum
Flat collector − Y-axis stepper motor bracket	L-type bracket for NEMA 17HE08-1004S	1	3	3	Local retailer	Aluminum
Flat collector − Neodymium magnet	Neodymium magnet	10	0.25	2.5	Link 3	Neodymium
Flat collector − M5x12 screw	M5x12 screw	28	0.07	1.95	Local retailer	Steel
Flat collector − M4x15 screw	M4x15 screw	36	0.07	2.52	Local retailer	Steel
Flat collector – M3x4 screw	M3x4 screw	10	0.07	0.7	Local retailer	Steel
Syringe pump − Syringe pump	Syringe pump NE-500	1	520	520	Link 7	Other
Syringe pump − Pump bracket plate 1	Acrylic plate 400 × 180 × 5 mm	1	5	5	Local retailer	Acrylic
Syringe pump − Pump bracket plate 2	Acrylic plate 160 × 130 × 3 mm	1	1	1	Local retailer	Acrylic
Syringe pump − Pump bracket plate 3	Acrylic plate 250 × 120 × 3 mm	1	1	1	Local retailer	Acrylic
Syringe pump − Pump Bracket aluminum Profile 1	Aluminum profile 120 × 40 × 20 mm	2	1.8	3.6	Link 1	Aluminum
Syringe pump − Pump Bracket aluminum Profile 2	Aluminum profile 150 × 40 × 20 mm	2	2.25	4.5	Link 1	Aluminum
Syringe pump − M5x12 screw	M5x12 screw	4	0.07	0.28	Local retailer	Steel
Syringe pump − M4x15 screw	M4x15 screw	16	0.07	1.12	Local retailer	Steel
Syringe pump − Syringe needle kit	Syringe needle kit	1	5.99	5.99	Link 8	Other
HVPS − HVPS AHVAC30KVR5MABT	HVPS AHVAC30KVR5MABT	1	559	559	Link 9	Other
HVPS − Banana plug for alligator clip cable	Banana plug for alligator clip cable	1	5.99	5.99	Link 10	Other
Control module − Laptop	Laptop	1	102	102	Link 11	Other
Control module − Arduino UNO	Arduino UNO	1	12	12	Local retailer	Other
Control module − Shiel CNC	Shield CNC V3	1	8	8	Link 12	Other
Control module − Driver A4988	Driver A4988	2	4	8	Link 13	Other
Control module − Stepper Motors Power supply	Stepper Motors Power supply	1	6.29	6.29	Link 14	Other
Total cost (USD)				1546.97		

## 5 Build instructions

### 5.1 Protective enclosure

**Fig. 7:**
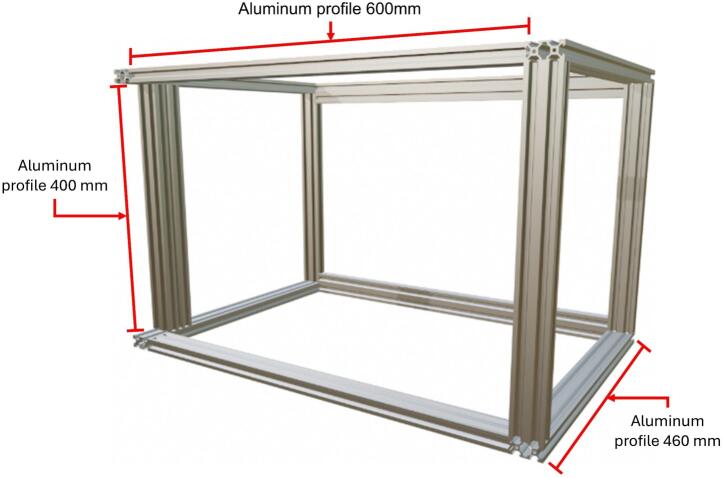
Structure of the protective enclosure.

The first step in constructing the electrospinning equipment is building the protective enclosure. Its primary function is to provide structural support for the internal components of the electrospinning system, such as the collector and syringe pump. The enclosure is composed of thirteen aluminum profiles measuring 40 mm × 20 mm, obtained from seven one-meter-long profiles. These profiles were cut to the following lengths: four at 600 mm, four at 460 mm, and five at 400 mm. The aluminum profiles are connected using two kits of twenty 25 mm × 26 mm zinc alloy L-brackets designed for T-slot grooves. The assembled structure of the protective enclosure is shown in Fig. 7.

### 5.2 Two-dimensional motion collector

The collector is built using two linear motion guide kits: one 300 mm in length and the other 400 mm. Each kit consists of:

● A T8 trapezoidal lead screw (300 mm or 400 mm long, 8 mm in diameter).
● Two bearings for the lead screw, serving as supports and enabling rotation along its axis.
● A T8 nut and T8 nut block, which together convert the screw's rotational motion into linear motion.
● Two flexible couplings to transmit motion from the motor to the lead screw.
● Two stainless steel sliding rods (300 mm or 400 mm long, 8 mm in diameter) equipped with four sliding blocks for linear motion.
● Four SK8 supports to secure the sliding rods (two per rod).

**Fig. 8:**
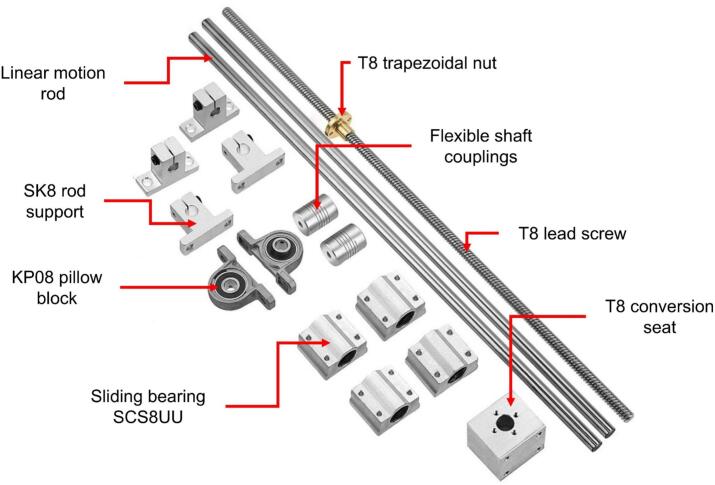
Components of the linear motion kit.

**Fig. 9:**
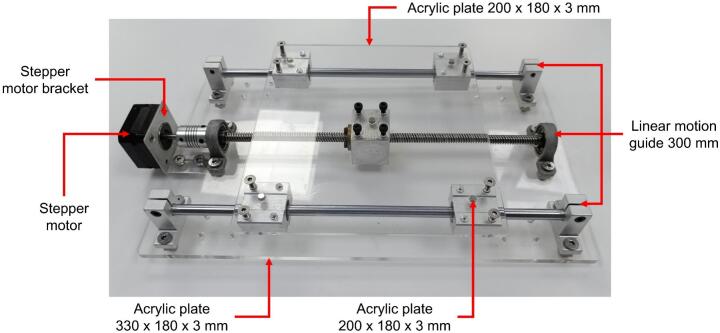
300 mm linear motion kit for vertical displacement.

**Fig. 10:**
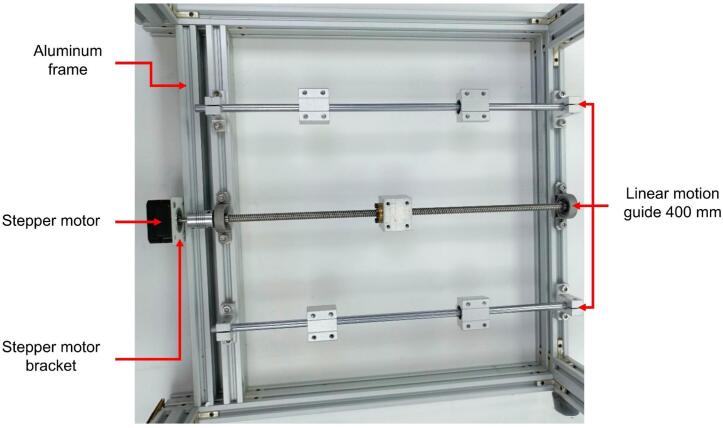
400 mm linear motion kit for horizontal displacement.

Except for the rods and the lead screw, all parts listed above are made of aluminum or brass alloys. The components of the linear motion guide kit are shown in Fig. 8. The collector plate is a flat, transparent acrylic sheet (200 mm × 200 mm × 3 mm) with a smooth surface on both sides. The upper surface is coated with aluminum foil for electrical conductivity. Five N35 neodymium magnets are attached to the back of the plate to enable quick removal for handling. The complete collector assembly is depicted in Fig. 9 and Fig. 10.

The collector’s body is made up of the following materials, which fulfill its structural and dynamic functions:

● One acrylic plate measuring 330 mm × 200 mm × 5 mm.
● One acrylic plate measuring 200 mm × 180 mm × 3 mm.
● One linear motion guide, 400 mm in length.
● One linear motion guide, 300 mm in length.
● Two stepper motors, model 17HE08-1004S.
● Two stepper motor mounts for model 17HE08-1004S.
● Ten N35 neodymium magnets.

The 300 mm linear motion guide is placed on the 330 mm × 200 mm × 5 mm acrylic plate along with one of the stepper motors, which is mounted using one of the motor mounts. The 200 mm × 180 mm × 3 mm acrylic plate is then attached on the top of the 300 mm guide. This plate is pre-fitted with five magnets on its upper side, which allow for quick and easy removal of the collector. The fully assembled mechanical setup can be seen in Fig. 9.

**Fig. 11:**
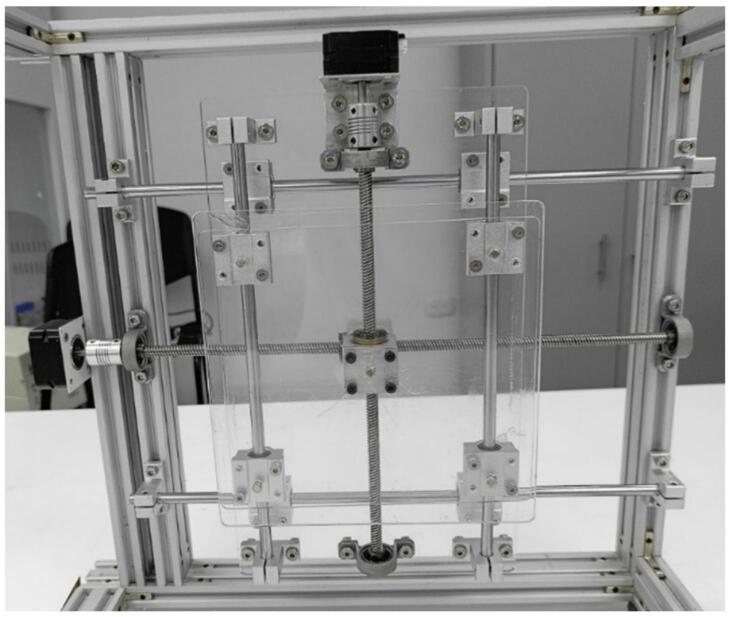
Mechanical structure of the collector.

The assembly in Fig. 9 is placed on the 400 mm linear motion guide, which was previously mounted on a rectangular frame made from extruded aluminum profiles that serve as the structural base for the electrospinning system. This creates the assembly shown in Fig. 10 and Fig. 11.

**Fig. 12:**
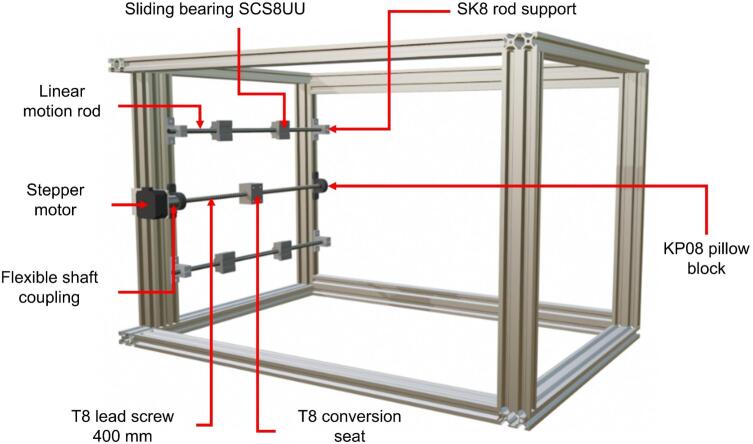
Assembly of the 400 mm linear motion guide.
● Attach the motor to one end of the lead screw using a coupling for NEMA 17 motors (5 mm–8 mm).

Given the components of the linear motion kits (see Fig. 8), the collector assembly proceeds as follows:

● Place the four supports for the sliding rods (two supports per rod) aligned opposite each other based on the specified measurements to secure the sliding rods at both ends.
● Attach two sliding blocks along each rod to enable free movement along the rod's length.
● Position the two bearings similarly to the sliding rod supports. These bearings are located between the two rods and serve as supports for the lead screw.
● Pass the lead screw through the T8 nut, which is attached to the sliding block, and secure it using M4 Bristol screws (see Fig. 12. Assembly of the 400 mm linear motion guide).

Once the main body of the protective enclosure is assembled (see Fig. 11), the collector structure is mounted. To achieve this, the 400 mm linear motion guide is installed along with one NEMA 17 stepper motor on the interior left side of the protective enclosure. It is positioned vertically relative to the top and moves along the X-axis (see Fig. 12). The guide is secured with M5 Bristol screws and M5 T-slot nuts for 20x20 profiles. The lead screw and motor are centered on the 400 mm aluminum profile, with a sliding rod placed 110 mm on either side of the screw. This setup results in the assembly shown in Fig. 12.

**Fig. 13:**
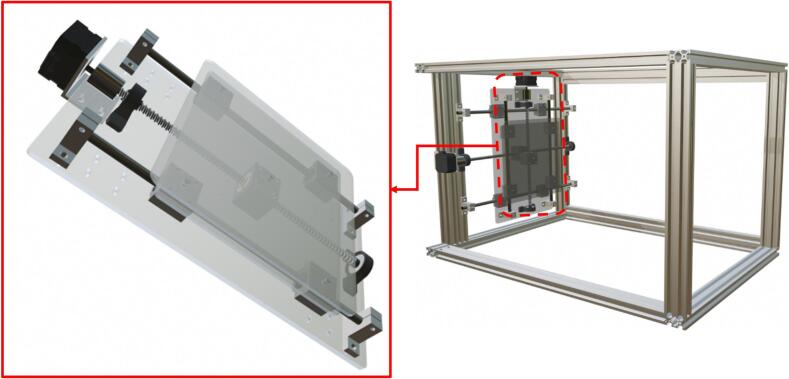
Assembly of the 300 mm linear motion guide and collector plate.

The 300 mm linear motion guide and the second stepper motor are then positioned on the 330 mm × 180 mm × 5 mm acrylic plate and secured with M4 Bristol screws (see Fig. 9). This assembly provides Y-axis (vertical) motion. Once assembled, this unit is bolted onto the 400 mm guide, previously installed in the electrospinning structure, with a 90° rotation relative to the larger guide. It is important to note that this assembly must be positioned with the motor at the top, as shown in Fig. 13.

To ensure easy and precise alignment, the acrylic plates used to connect the linear motion guides feature custom-drilled holes made using a laser cutter. The resulting electrospinning equipment resembles a horizontal 3D printer, with the distinct feature that the needle remains stationary while the collector plate moves in the XY plane.

### 5.3 Syringe pump base

Once the collector is assembled, the syringe pump is mounted inside the protective enclosure. A custom base was designed for this purpose using the following materials:

● One 400 mm × 180 mm × 5 mm acrylic plate.
● One 160 mm × 130 mm × 3 mm acrylic plate.
● One 250 mm × 120 mm × 3 mm acrylic plate.
● Two aluminum profiles measuring 40 mm × 20 mm × 120 mm.
● Two aluminum profiles measuring 40 mm × 20 mm × 150 mm.

**Fig. 14:**
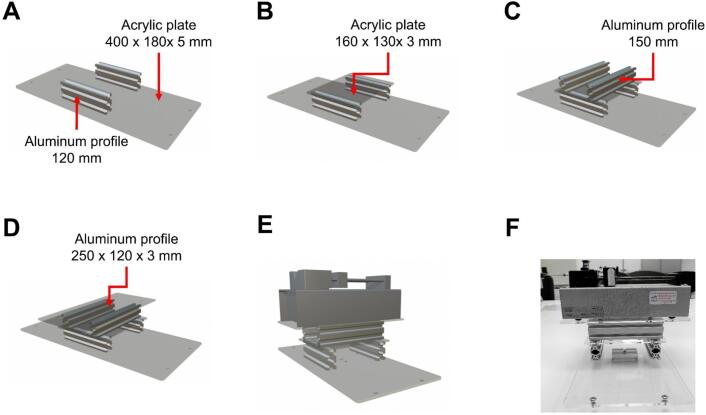
Assembly process of the pump and its structural base. A) Base plate with two aluminum profiles. B) Addition of small acrylic plate. C) Installation of upper aluminum profiles. D) Mounting plate for syringe pump. E) Pump integration. F) Final assembled support with syringe pump.

**Fig. 15:**
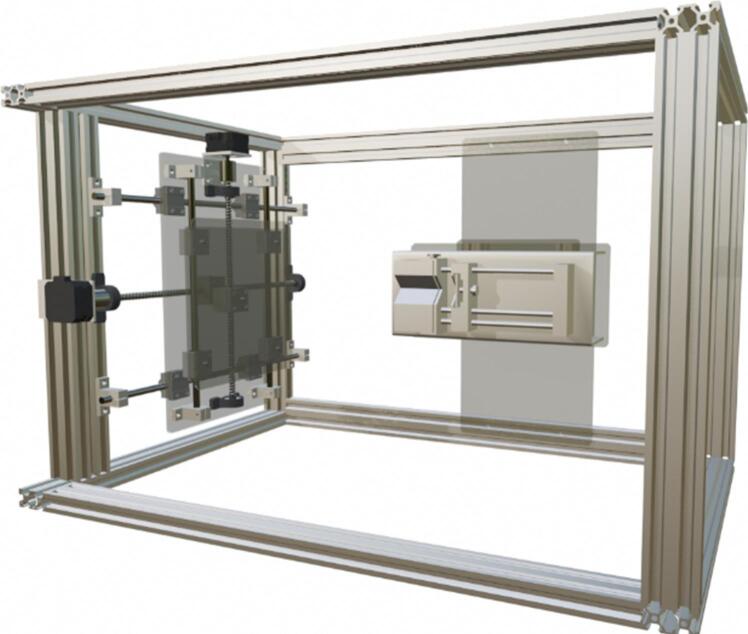
Electrospinning structure with the attached syringe pump.

The 400 mm × 180 mm × 5 mm acrylic plate serves as the direct connection between the pump's support structure and the internal framework of the electrospinning equipment. On this plate, two aluminum profiles measuring 120 mm are mounted (see Fig. 14A). The 160 mm × 130 mm acrylic plate is then placed on top of these profiles as a connecting layer (Fig. 14B). Two additional aluminum profiles, each 150 mm long, are installed on top of the connecting plate (Fig. 14C). Finally, the 250 mm × 120 mm acrylic plate is positioned at the top of the assembly to hold the syringe pump (Fig. 14D–F). This multi-layered support structure provides the necessary height for the needle to align with the center of the collector, as shown in Fig. 15.

**Fig. 16:**
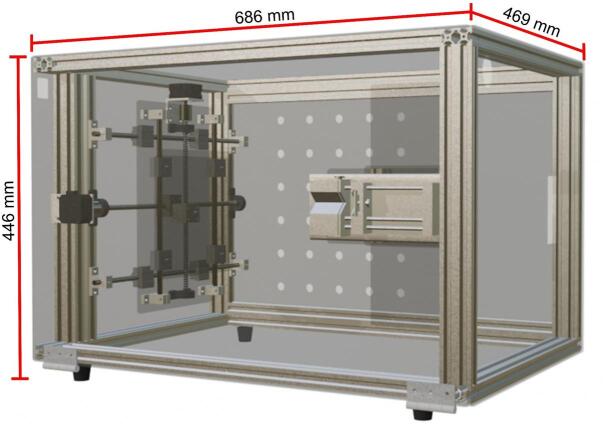
Electrospinning equipment.

As the next step, the exterior panels of the protective enclosure are assembled along with the rubber feet (see Fig. 16). The panels are made of 3 mm thick acrylic. The top and bottom panels measure 680 mm × 460 mm, while the left and right side panels measure 466 mm × 443 mm. Finally, the front and back panels measure 680 mm × 443 mm. It is worth noting that all acrylic plates and panels used in the assembly of the electrospinning equipment were custom-cut using a laser cutter.

As the final step, the control module is assembled. The CNC Shield is mounted on top of the Arduino UNO board by pressing the two components together until they are securely connected as a single unit. Next, the two drivers (one for each motor) are installed in the designated slots on the CNC Shield, along with the wiring responsible for transmitting current to the motors, which is positioned next to each driver. The drivers are equipped with a passive aluminum heat sink included with the components to prevent overheating.

**Fig. 17:**
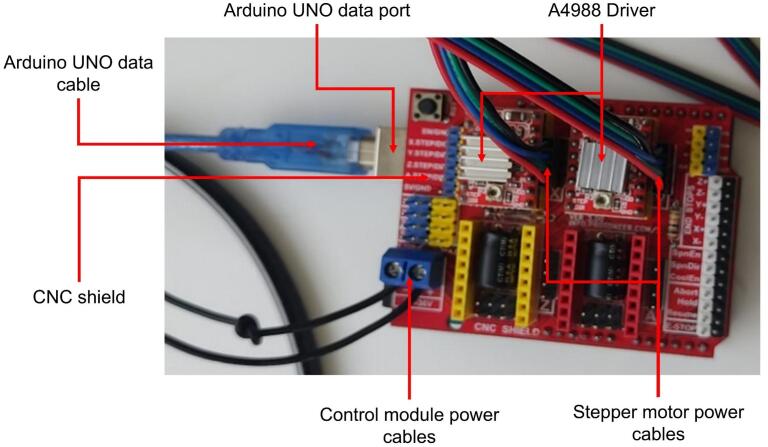
Control module of the electrospinning equipment.

**Fig. 18:**
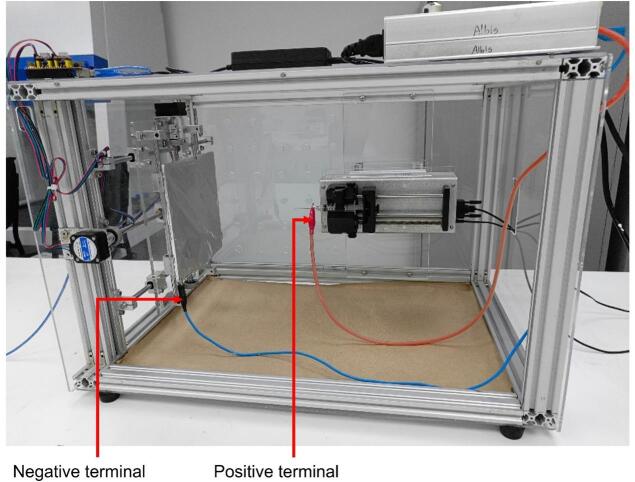
Electrospinning equipment fully assembled and operational.

Finally, the power cables are connected to the CNC Shield, and the data transmission cable is connected to the Arduino UNO board. This assembly is shown in Fig. 17. Once all parts of the electrospinning equipment are assembled and ready, the electrical connections for all components are completed, marking the final step in the construction of the electrospinning system. This is illustrated in Fig. 18.

## 6 Operation instructions

To properly operate the electrospinning equipment, the following precautions and recommendations should be observed:

● Ensure that all electrical connections are secure and in good condition.
● Use the equipment in a well-ventilated and dry area, or inside an exhaust hood when handling chemical reagents during the preparation, use, and disposal of polymer solutions in the electrospinning process.
● Always wear gloves, safety goggles, a protective mask, a lab coat covering the torso and arms, and closed shoes (preferably with rubber soles to reduce the risk of electrocution).
● Only trained personnel should operate the electrospinning equipment.

It is essential to handle the equipment and chemical reagents with the appropriate safety measures. The following steps should be followed to operate the equipment:

Connect the following components to the power supply: computer, stepper motors, syringe pump, and high-voltage power supply.
Set the appropriate polymer flow rate on the syringe pump using the SyringePumpPro software.
Turn on the computer and connect the Arduino UNO board with the CNC Shield to the computer.
Launch the UGS software and load the corresponding G-code (.ngc) file to configure the collector plate's movements. Additionally, set the motor speed and process duration in the G-code file.
Prepare the collector plate by covering it with a layer of aluminum foil.
Turn on the exhaust hood and prepare the polymer solution inside it, ensuring the correct proportions. Once ready, fill a 10 mL syringe with the appropriate amount of solution and attach a needle of the required gauge.
Secure the syringe filled with polymer into the syringe pump supports and fasten it using the provided knobs.
Connect the high-voltage supply terminals: the red cable (positive terminal) to the syringe needle and the black cable (negative terminal) to the bottom-right corner of the collector plate (see Fig. 18).
Close the electrospinning enclosure and activate the syringe pump using the foot pedal.
Start the motor movement pattern using the UGS software. Once the collector's motion is initiated, promptly turn on the high-voltage power supply and adjust the voltage to the required value.
After the electrospinning process is complete, stop the syringe pump using its foot pedal. Set the voltage on the high-voltage power supply to 0 kV using the potentiometer and turn off the supply. If the motor movement has not stopped automatically, manually halt it using the UGS software.
Disconnect the high-voltage power supply terminals. At the same time, remove the syringe and detach the needle.
Carefully place the needle into a container filled with the solvent used to prepare the polymer solution, then dispose of the syringe.
Allow the membrane to rest inside the equipment for 24 h before removing the collector plate to handle the membrane.

## 7 Validation and characterization

Operating electrospinning equipment safely requires strict compliance with safety protocols due to the use of high-voltage components. Therefore, only trained laboratory staff should utilize the system. Thereby, future users must review and understand the guidelines established in their corresponding user guide. It is essential that, before using any component, the user inspect the device and confirm that all electrical and mechanical connections are secure and undamaged. Furthermore, the entire system must be operated inside a fume hood to minimize exposure to potentially toxic vapors and reduce the risk of electric shock. To avoid electrocution hazards, it is also essential to maintain a dry environment within the hood.

During polymer solution preparation and the electrospinning process, appropriate personal protective equipment (PPE), including safety goggles, gloves, and protective masks, must always be worn. In the event of an emergency, the high-voltage power supply must be immediately shut off, and all cables disconnected before any physical contact with the equipment. Therefore, all operators should be familiar with the location of the power switches and emergency procedures established in the laboratory.

Routine maintenance is also crucial for ensuring reliable equipment performance over time. Regular visual inspections should be carried out to detect signs of mechanical wear, corrosion, or damage, especially in key areas such as the syringe holder, wiring, and linear motion guides. To maintain the smooth mechanical function of moving components, such as rails and motors, should be periodically lubricated using 3D printer grease. If lubrication is repeated, residual grease must be cleaned with a degreaser to avoid operational blockages.

Regarding the collector surface, the aluminum foil lining the collector plate should be replaced regularly, as accumulation or degradation can affect membrane uniformity and removal efficiency. Likewise, electrospinning needles must be carefully cleaned after each use by soaking them in the same solvent used for polymer preparation, ensuring to remove any residual material that can obstruct future runs. On the other hand, to preserve the structural integrity of the electrospinning, abrasive solvents or cleaning products should be avoided, especially on acrylic or electrical components. Instead, a soft and dry cloth is recommended for cleaning the external surfaces of the system. By following these safety, maintenance, and operational guidelines, users can ensure consistent performance, user protection, and extended equipment lifespan.

While these operational measures ensure safety and reliability, it is also essential to address the system's inherent limitations and scalability challenges. Electrospinning with a flat collector plate is common in laboratories due to its simplicity and low cost. However, it has limitations, including limited control over fiber alignment, fiber deposition, uniformity, and scalability [30,31].

A flat plate collector typically gathers fiber from a single point source, like a needle or nozzle. This restricts the fiber deposition speed compared to systems with multiple nozzles or alternative collector designs, like drum collectors [32]. Furthermore, without constant movement of the collecting surface, the deposition process slows down, leading to lower fabrication speeds [33].

Furthermore, on a flat collector, fibers tend to deposit randomly, preventing the formation of aligned or patterned fiber mats [30]. Additionally, the electric field intensity is higher at the edges, which causes preferential fiber accumulation zones at the borders and results in thickness variation across the membrane [34].

These challenges led us to create a plate collector that can sustain consistent motion in the XY plane for this project. The movable plate collector allows our electrospinning process to produce membranes with uniform thickness by reducing buildup on the collector plate through movement. Additionally, it increases the deposition rate, resulting in quicker and more cost-effective production.

Although the design presented in this study addresses several issues, scalability remains a challenge. A natural solution is to increase the size of the collector, which should theoretically boost throughput; however, this also complicates maintaining a uniform electric field across the surface, resulting in uneven fiber deposition [35]. It is worth noting that the scalability problems affect even standard electrospinning designs, such as drum collectors, which enable the collection of a large amount of fibers but with inconsistent thickness [36].

To address these limitations, several alternative strategies have been developed to enhance scalability, fiber quality, and uniform thickness [37]. One of these strategies is the denominated roll-to-roll (R2R), which enables continuous membrane production. This process involves a supply roll that feeds a steady strip of substrate material into the electrospinning zone. The substrate then moves at a controlled speed across the collector area and is finally wound onto a take-up roll [38]. An alternative approach uses multi-nozzle configurations to boost throughput by producing multiple jets at once, improving the membrane recollection, and creating membranes with regular thickness [39,40]. These setups need precise nozzle spacing, careful voltage adjustments, or auxiliary flow fields to reduce interference and ensure uniform deposition [41].

To evaluate the system’s performance, a polymer solution was prepared using virgin polystyrene (PS) and dimethylacetamide (DMAc). In this study, polystyrene (PS) was selected for this electrospinning validation because of its consistent electrospinnability, chemical stability, mechanical strength, and affordability. PS can dissolve in various organic solvents such as DMF, DMAc, acetone, and others [42,43], enabling the production of uniform fibers with minimal bead formation, which makes it suitable for reproducible testing [44]. Additionally, its chemically inert nature helps the fibers maintain their morphology during characterization, ensuring consistent validation results [45,46]. Another significant advantage is its low cost, making it a practical choice for initial validation experiments [47].

Furthermore, PS is compatible with a wide range of polymers such as PCL, PA6, or PPO [48,49]. These combinations enhance mechanical and surface properties, such as tensile strength, flexibility, hydrophobicity, and permeability [50]. Due to these benefits, PS is an effective polymer for validation and is suitable for integration into various electrospinning setups and applications [51].

The PS and DMAc were mixed in a weight-to-volume ratio of 20 % w/v for 24 h. The conductivity of the solution was then adjusted to approximately 7.3 µS/cm using pulverized sodium chloride [52]. Next, 4 mL of the solution were drawn into a 10 mL syringe equipped with a blunt tip steel needle (24 G). The polymer solution was injected at a pumping rate of 1 mL/h, with a distance of 12.5 cm between the needle and the collector. The entire process lasted approximately 3 h and 30 min, during which the system was operated at four different voltages (14 kV, 16 kV, 18 kV, and 20 kV) to study the morphology of the membranes produced under varying voltage conditions.

### 7.1 SEM images

**Fig. 19:**
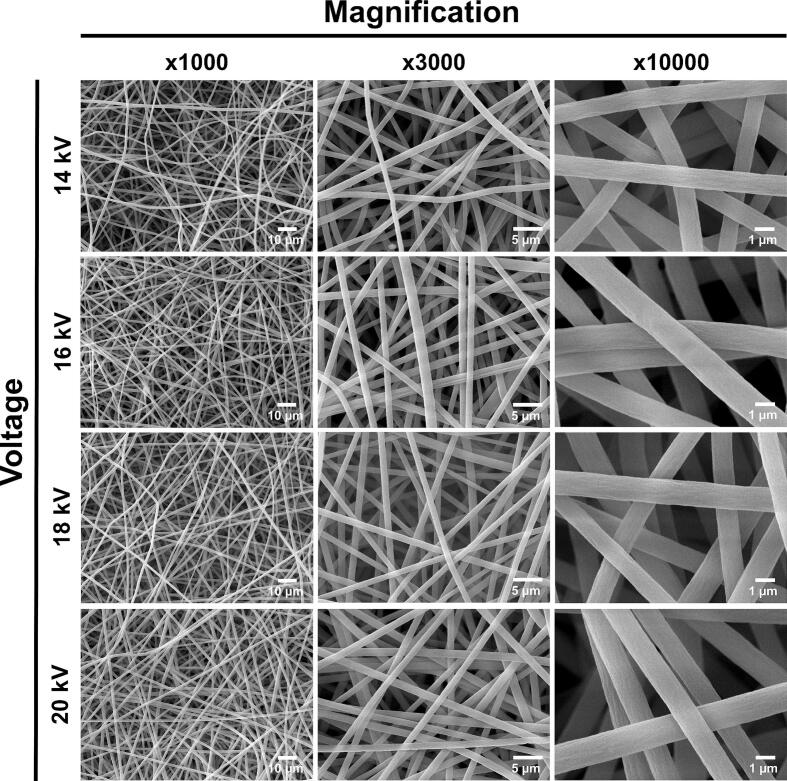
SEM images of PS-based nanofibers from membranes obtained at various voltages, 14 kV, 16 kV, 18 kV, and 20 kV, and magnifications, x1000, x3000, and x10,000.

Fig. 19 presents SEM images of the membranes obtained at 14 kV, 16 kV, 18 kV, and 20 kV, captured at magnifications of 1000x, 3000x, and 10000x using a JEOL JSM 6490 LV scanning electron microscope. These SEM images were analyzed using the ImageJ software to determine the average fiber diameter and pore size of the electrospun membranes.

### 7.2 Fiber diameter

**Fig. 20:**
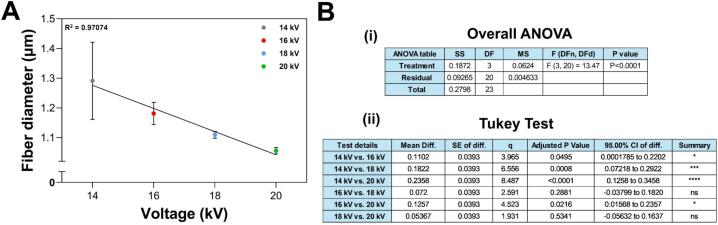
Fiber diameter variation in each membrane (A) as a function of the applied voltage. (B) Error analysis for fiber diameter: (i) ANOVA and (ii) Tukey test. One-way ANOVA showed that applied voltage significantly affects fiber diameter (F = 13.47, p < 0.0001). Linear regression revealed a strong negative correlation (R^2^ = 0.97), indicating that increasing voltage results in smaller fibers. The Tukey test showed significant differences among the voltage ranges of 14–16 kV, 14–18 kV, 14–20 kV, and 16–20 kV. In contrast, differences between similar voltage levels, 16–18 kV, and 18–20 kV, were not statistically significant. These results underscore that voltage plays a crucial role in influencing fiber diameter. * p < 0.05, *** p < 0.001, **** p < 0.0001.

Fig. 19 reveals that the fibers obtained exhibit a predominantly uniform and continuous appearance across all voltages. However, Fig. 19, Fig. 19 show small beads on the fibers produced at 14 kV, which are associated with insufficient voltage [53]. Fig. 20 illustrates an inverse relationship between the average fiber diameter and the applied voltage, indicating that higher voltages result in thinner fibers [54,55]. Additionally, as the voltage increases, the variability in fiber diameters within the same membrane, represented by error bars, decreases significantly. Thus, at 20 kV, the fibers are more uniform across the entire membrane compared to those produced at 14 kV, which display greater diameter variability. The average diameters range from 1.3 µm at the lowest voltage to 1.05 µm at the highest voltage. These submicrometer-scale diameters are influenced by factors such as the polymer and solvent used (as well as their proportions), solution conductivity, and manufacturing variables, including voltage, needle-to-collector distance, relative humidity, polymer flow rate, and syringe pump precision [[56], [57], [58]]. Especially, the latter plays an important role in fiber quality, as a consistent Taylor cone and a uniform polymer jet producing homogeneous diameters depend on flow rate stability [[59], [60], [61]]. Therefore, alterations in the flow rate can cause irregularities in the polymer jet, which translates into a high variability in fiber diameter distribution, surface morphology defects, and bead formation [62,63]. Likewise, higher syringe pump flow rates tend to produce thicker fibers, since they can cause an incomplete evaporation of the solvent before fiber deposition in the collector [60,62]; while lower flow rates can produce thinner fibers but also cause instability of the polymer jet, which increase the formation of beads [60]. In our low-cost bidirectional plate model, we carefully calibrated the syringe pump, which allowed us to maintain a stable flow rate during the process and contributed to obtain consistent fiber diameters with low variability, as reported in our results.

### 7.3 Contact angle

**Fig. 21:**
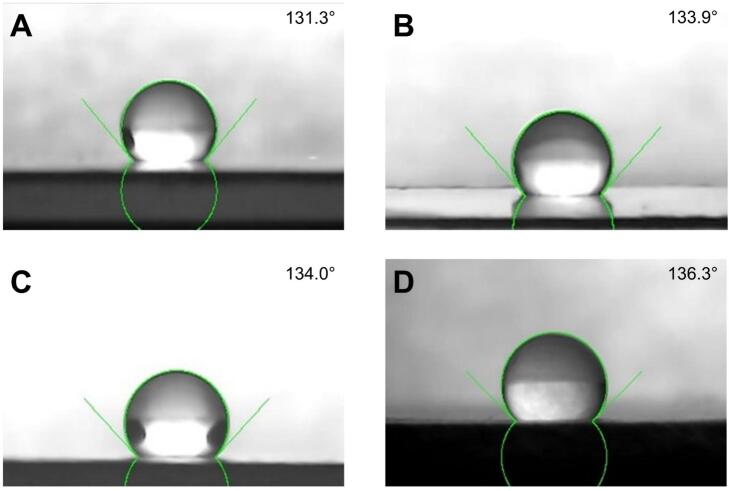
Contact angle measurements for the membranes obtained at: (A) 14 kV, (B) 16 kV, (C) 18 kV, and (D) 20 kV.

**Fig. 22:**
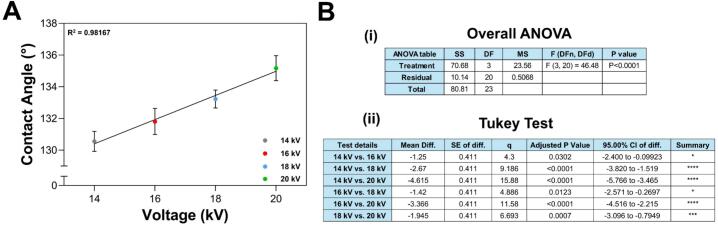
Contact angle variation (A) as a function of the applied voltage. (B) Error analysis for contact angle: (i) ANOVA and (ii) Tukey test. One-way ANOVA showed that applied voltage significantly affects the contact angle of the membranes (F = 46.48, p < 0.0001). Additionally, linear regression revealed a strong positive correlation (R^2^ = 0.98), suggesting that increasing voltage results in a larger contact angle. The Tukey test showed significant differences between all pairs of voltage levels. These results underscore that voltage plays a crucial role in tuning the surface wettability. * p < 0.05, *** p < 0.001, **** p < 0.0001.

Contact angle measurements were performed by placing a drop of distilled water on a membrane and capturing its shadow using a Hayear 150× camera with backlighting. The resulting images were processed using ImageJ software to determine the contact angle and assess the hydrophobicity of the membranes. Fig. 21 displays the contact angle measurements for membranes produced at 14 kV, 16 kV, 18 kV, and 20 kV. As shown in Fig. 22, there is a direct relationship between the applied voltage and the contact angle, suggesting that higher manufacturing voltages result in more hydrophobic membranes [64]. This can be attributed to the formation of thinner fibers at higher voltages, which increase the number of layers in the membrane and enhance its ability to trap air, thus increasing hydrophobicity [65]. Unlike fiber diameter, the variability in contact angles among membranes produced at the same voltage does not appear to correlate directly with the applied voltage.

### 7.4 Pore size and surface area

**Fig. 23:**
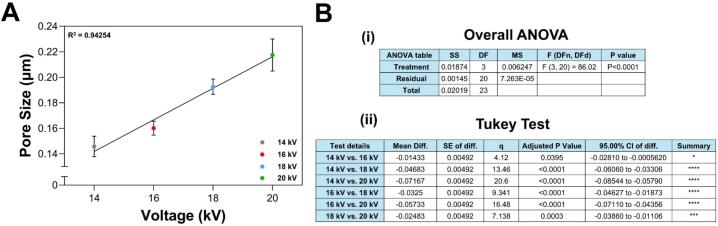
Pore size variation of the membranes (A) as a function of the applied voltage. (B) Error analysis for pore size: (i) ANOVA and (ii) Tukey test. One-way ANOVA revealed that applied voltage significantly affects pore size (F = 86, p < 0.0001). Linear regression revealed a strong positive correlation (R^2^ = 0.94), indicating that increasing voltage results in a large pore size. The Tukey test revealed significant differences between all voltage level pairs. These findings highlight the importance of voltage in modulating pore size. * p < 0.05, *** p < 0.001, **** p < 0.0001.

Fig. 23 shows that pore size in the membranes is directly related to the applied voltage. Higher manufacturing voltages result in larger pores. This trend aligns with the observed relationship between fiber diameter and voltage. As higher voltages produce thinner fibers, they occupy less space, leading to larger gaps between fibers as the voltage increases. This supports the results obtained for fiber diameter [55]. Additionally, variability in pore size among membranes produced at the same voltage increases significantly with higher voltages. As a result, lower voltages yield more uniform pore sizes, while higher voltages produce greater differences in pore size.

### 7.5 Porosity and thickness

**Fig. 24:**
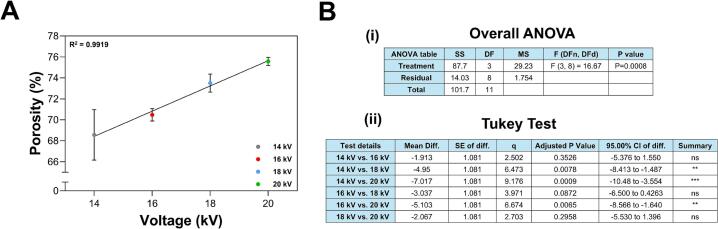
Porosity percentage (A) as a function of the applied voltage. (B) Error analysis for porosity: (i) ANOVA and (ii) Tukey test. One-way ANOVA indicated that applied voltage significantly influences membrane porosity (F = 16.67, p < 0.0008). Linear regression demonstrated a strong positive relationship (R^2^ = 0.99), implying that increasing voltage boosts porosity. The Tukey test showed significant differences between 14–18 kV, 14–20 kV and 16–20 kV, whereas other pairs were not statistically different. These results emphasize voltage as a critical factor in controlling porosity, with the most pronounced differences seen between the lowest and highest voltage levels. * p < 0.05, *** p < 0.001, **** p < 0.0001.

Porosity measurements were performed using a gravimetric test. For this, three 2 cm × 3 cm samples were cut from the membranes, weighed dry, and then immersed in isopropanol. Excess isopropanol was removed with Whatman filter paper, and the samples were placed on an analytical balance (Optika Italy model B214AJ) to measure weight changes over time until all the isopropanol evaporated. The porosity percentage of the membranes was calculated based on these measurements. Fig. 24 shows a direct correlation between the porosity percentage of the membranes and the applied manufacturing voltage. As the voltage increases, so does the porosity percentage. Membranes fabricated at 14 kV exhibit greater variability in porosity compared to those produced at higher voltages.

**Fig. 25:**
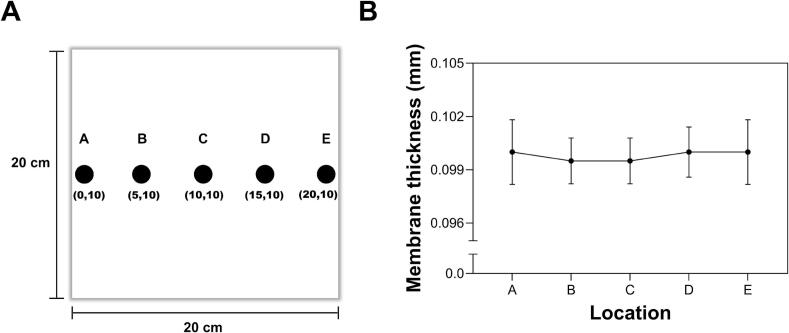
Nanofibrous membrane thickness across at (A) different locations on the surface and (B) its values.

Membrane thickness was measured using a Mitutoyo 0–25 mm digital external micrometer, with 20 measurements taken across the membrane surface. Tests were conducted at different collector speeds to determine the speed range that produced the most uniform membranes with an approximate thickness of 0.1 mm. Initial collector’s motor speeds ranged from 2000 to 3000 mm/min, producing membranes with an average thickness of 0.133 ± 0.0576 mm. Increasing the speed range to 3000–4000 mm/min resulted in an average thickness of 0.109 ± 0.0306 mm. Finally, a motor speed of 5000 mm/min yielded membranes with an average thickness of 0.100 ± 0.0014 mm across the surface, as shown in Fig. 25. Notably, this thickness was independent of the applied voltage, contributing to membrane thickness uniformity.

To validate these results, our proposed model was compared with previous works in literature. For instance, a recent scaled-up electrospinning [39] consisting of multiple needles in series and using a simple static flat collector reported membrane thickness values ranging from ∼0.004 to ∼0.014 mm across the deposition surface. After implementation of auxiliary electrodes, membrane thickness values ranged from ∼0.012 to ∼0.014 mm. Additional PVDF-HFP electrospun membranes were fabricated using a 20 % (w/w) PVDF-HFP solution in DMF/acetone (2.3:1, w/w) at a flow rate of 2.5 mL/h. The electrospinning setup consisted of a rotating drum collector (Hanmark; width: 12 cm, diameter: 4.8 cm) mounted on a laterally translating platform (Falco). The resulting membranes exhibited lower thickness uniformity with thickness values ranging from approximately 0.043 to 0.130 mm. In contrast, our bidirectional collector produced membranes with an average thickness of 0.1 mm with a standard deviation of 0.0014 mm, measured at five evenly distributed locations across the collector surface, as illustrated in Fig. 25. This represents a variation of only 1.4 % in comparison to the multi-needle setup, highlighting the performance achieved by our proposal in terms of membrane thickness compared to possible commercial electrospinning configurations, even with the aid of auxiliary electrodes. Similar challenges related to thickness inconsistencies have been described in other studies [23,66,67] in which alternative designs of stationary flat collectors have been implemented, such as discs, wire drums, parallel bars, polygonal collectors, and drum collectors, the latter being one of the most affected by the lack of uniformity and consistency in membrane thickness [23,66].

Furthermore, fiber diameter and contact angle values reported using the bidirectional flat plate are consistent with previous electrospinning reports. For example, a polystyrene-based [68] electrospun nanofibers study reported an average value of 1.214 µm at 15 % w/v of polystyrene. This result is consistent with the outcomes provided by the proposed model, which ranged from 1.3 to 1.05 µm. Another polystyrene-based [69] approach exhibited higher fiber diameter values (∼12.7 µm) and contact angles that ranged from ∼95° to ∼155°, which are coherent with the obtained using the bidirectional plate (130.5°–135.5°). These findings demonstrated that the low-cost bidirectional plate model was capable of replicating and, in some cases, improving upon the results described in the literature for high-cost or potentially scalable electrospinning studies [66]. This performance was possible given its greater range and movement in all directions, which allowed for better deposition and homogeneity across the membrane surface. These outcomes are relevant when comparing the difference in price, higher than $4000 USD, and accessories offered by high-cost and scalable electrospinning, such as environmental chambers with temperature and humidity control.

Unlike our polystyrene-based system, which is optimized to provide consistency in the bulk membrane thickness for membrane distillation, most commercial and high-cost configurations are primarily optimized to produce nanofibers with specific diameters. This design rationale is related to the wide range of potential applications offered by nanofibers extracted from electrospun membranes. Within this wide range, energy applications stand out, such as photothermal technologies for separation and adsorption [70]; environmental applications, such as pesticide and dye removal [71,72] and manufacture of nano air filters [38,66]; and biomedical applications, such as tissue regeneration [73,74] and scaffolds within hydrogels to support three-dimensional cell culture, the latter being one of the most promising applications, given its ability to be modified through crosslinkers for tunable applications in the biomedical field [56,75].

## Ethics statements

No ethical approval was required.

## CRediT authorship contribution statement

**Jhonatan A. Gutierrez-Rivera:** Writing – review & editing, Writing – original draft, Validation, Software, Investigation, Formal analysis, Data curation, Conceptualization. **Andres F. Roca-Arroyo:** Writing – review & editing, Writing – original draft, Validation, Software, Investigation, Formal analysis, Data curation, Conceptualization. **David A. Castilla-Casadiego:** Writing – review & editing, Writing – original draft, Visualization, Supervision, Resources, Funding acquisition. **Alberto Albis:** Writing – review & editing, Writing – original draft, Visualization, Supervision, Resources, Funding acquisition.

## Funding

This research was funded by the 10.13039/100000057National Institute of General Medical Sciences of the 10.13039/100000002National Institutes of Health under grant number R00GM151459.

## Declaration of competing interest

The authors declare that they have no known competing financial interests or personal relationships that could have appeared to influence the work reported in this paper.
